# Advances in Transepithelial Photorefractive Keratectomy versus Laser-Assisted In Situ Keratomileusis

**DOI:** 10.3390/diagnostics14050481

**Published:** 2024-02-23

**Authors:** Paul Filip Curcă, Cătălina Ioana Tătaru, George Sima, Marian Burcea, Călin Petru Tătaru

**Affiliations:** 1 Clinical Department of Ophthalmology, “Carol Davila” University of Medicine and Pharmacy, 020021 Bucharest, Romania; filipcurca@yahoo.com (P.F.C.); george.sima27@gmail.com (G.S.); mnburcea@gmail.com (M.B.); calinpetrutataru@yahoo.com (C.P.T.); 2 Department of Ophthalmology, Clinical Hospital for Ophthalmological Emergencies, 010464 Bucharest, Romania; 3 Alcor Clinic, 030829 Bucharest, Romania

**Keywords:** Trans-PRK, LASIK, versus, transepithelial photorefractive keratomileusis

## Abstract

(1) Background: Laser-assisted refractive surgery is a safe and effective surgical correction of refractive error. For most patients, both the newer Trans-PRK and the established LASIK technique can produce the required surgical correction, sparking the question of which technique should be opted for. (2) Methods: The study prospectively evaluated 121 patients (230 eyes) for at least one month postoperatively; 66 patients (126 eyes) and 45 patients (85 eyes) returned for 6 months and 1 year follow-up. (3) Results: No statistical difference was recorded at 1 week or 1 month post-operation. At 6 months, a difference was found for spherical diopters (Trans-PRK −0.0476 ± 0.7012 versus FS-LASIK +0.425 ± 0.874, *p* = 0.004) and spherical equivalent (Trans-PRK −0.1994 ± 0.0294 versus FS-LASIK +0.225 ± 0.646, *p* = 0.025) but not for CYL D (Trans-PRK −0.3036 ± 0.5251 versus FS-LASIK −0.4 ± 0.820, *p* = 0.499). Uncorrected visual acuity was better for Trans-PRK 6 months post-operation (UCVA logMAR 0.02523 versus 0.0768 logMAR; *p* = 0.015 logMAR). At 1-year, Trans-PRK was favored for spherical diopters (Trans-PRK −0.0294 ± 0.6493 versus FS-LASIK +0.646 ± 0.909, *p* < 0.001) and spherical equivalent (Trans-PRK −0.218 ± 0.784 versus FS-LASIK 0.372 ± 1.08, *p* = 0.007). Overall speed in visual recovery, variance of results and surgically induced astigmatism were in favor of Trans-PRK. (4) Conclusions: The study reported improvements for Trans-PRK patients, with both techniques found to be safe and effective.

## 1. Introduction

Laser-assisted refractive surgery is widely recognized as a safe and effective surgical procedure [1] which uses ablation of the corneal tissue to modify the corneal refractive profile, obtaining surgical correction of the refractive error (myopia, hyperopia, astigmatism, and presbyopia) [1]. Several techniques are currently in use. Laser-assisted in situ keratomileusis (LASIK) first involves the creation of a corneal flap using femtosecond near-infrared laser [2], followed by corneal stromal ablation using ultraviolet excimer laser beneath the corneal flap [1,2]. Surface ablation procedures such as photorefractive keratomileusis (PRK) variants involve the removal of the epithelium, followed by ablation of Bowman’s layer and the anterior corneal tissue [1]. In the transepithelial photorefractive keratomileusis (Trans-PRK) technique, both the initial surface ablation and the subsequent stromal ablation are created using the same high-precision UV excimer laser [3,4]. Comparatively, excepting mostly hyperopia, high myopia, and astigmatism, both the newer Trans-PRK or the established LASIK technique can produce the required surgical correction, sparking the question of which technique should be opted for.

**Figure 1:**
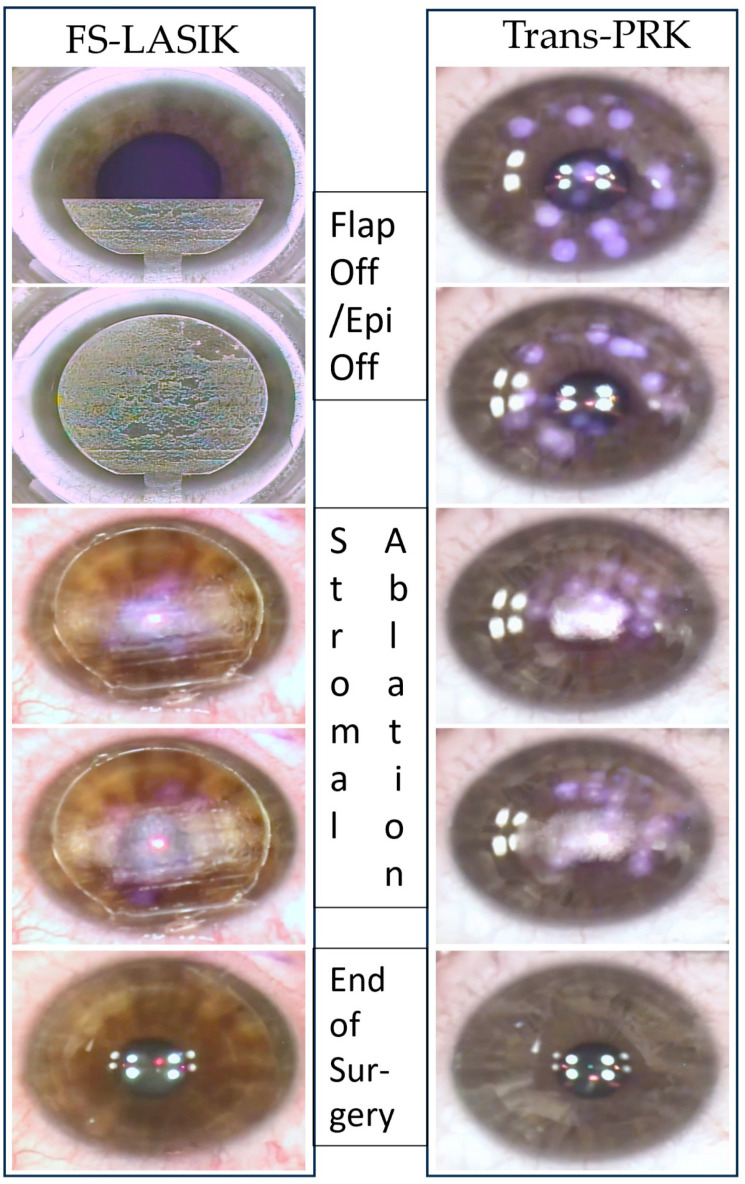
Clinical images from the study exemplifying the surgical steps of femtosecond-laser-assisted in situ keratomileusis (FS-LASIK) versus transepithelial photorefractive keratectomy (Trans-PRK). For FS-LASIK, the femtosecond laser (Wavelight^®^ FS200^®^ 50 Hz, 193 nm) first creates a corneal flap. The completed flap is set aside exposing the corneal stroma. A different ultraviolet (UV) excimer laser (Wavelight^®^ EX500^®^ 500 Hz) produces the stromal ablation for refractive correction. The flap is repositioned, and surgery is ended. In Trans-PRK, the procedure is entirely completed using the UV excimer laser. After ablating through the epithelium and epithelial basement membrane access to the stroma is gained (Epithelium/Epi off). Stromal ablation follows producing the desired refractive correction. The excised stroma is gently set aside using an atraumatic surgical sponge. Mitomycin-C 0.02% is applied using the sponge at the end of the surgery.

LASIK surgery is highly advantageous in its corrective potential: as long as a sufficiently safe thickness of corneal stroma remains post-operatively, LASIK can be used to quickly correct high myopia up to −18.5 diopters (D) [5], high hyperopia (+8D) [6], and associated high astigmatism [7]. Biomechanically LASIK requires the creation of the corneal flap, which once created can reduce the biomechanical strength of the cornea, increasing risk of corneal ectasia [1], and is dependent on the healing and corneal reintegration process of the individual patient (Figure 1). Mismatch of the femtosecond-cut undersurface of the flap with the ablated corneal stroma and diffraction from the femtosecond laser grating pattern [8] could lead to subjective photic phenomena, such as “rainbow glare” [8] or corneal haze, and scarring. 

Trans-PRK has a lower corrective power and longer surgical ablation time, however, avoids the biomechanical disadvantage of the flap. In the process the epithelium, epithelial basement membrane (which plays a central function in the process of corneal epithelial wound healing [9]) and Bowman’s layer [1] are all ablated (Figure 1). As such, Trans-PRK also depends on the individual’s epithelial healing process, which can incur delayed healing, leading to patient discomfort, dry eye disease, or healing abnormalities such as subepithelial fibrosis and corneal haze. The latter can be mostly mitigated by the application of low-dose topical mitomycin-C (ranging from 0.02% to 0.04%) [1].

## 2. Materials and Methods

### 2.1. Study Design and Aim

For the prospective study, 121 patients (total 230 eyes) seeking laser-assisted refractive surgery at the Alcor Clinic (“Clinica Alcor”, Bucharest, Romania) via Trans-PRK (87 patients—167 eyes) or FS-LASIK (34 patients—63 eyes) in the 2019–2022 time period were selected. The study aimed to compare overall results of Trans-PRK and FS-LASIK in a clinical, non-randomized context, selected based upon the complete preoperatory assessment as the recommended surgical technique in accordance with the patient’s pathology and was selected independently of the study. Patient selection criteria for the study included the following: age at least 18 years old, informed consent agreement for study participation, presence of the following refractive pathology: myopia, hyperopia, myopic or hyperopic astigmatism, and surgical indication for laser-assisted refractive surgery. Preoperative screening required stable refraction and keratometry measurements for at least 1 year pre-operative, absence of refractive-surgery contraindications such as unstable corneal ectasia and excessively thin corneas or the presence of other contraindications such as corneal dystrophy, cataract or congenital cataract, glaucoma, and significant retinal pathology. Patients who underwent other refractive surgery procedures, such as older interventions with laser-assisted refractive surgery, phakic implants, or corneal intrastromal ring implants, were excluded. The surgical technique was selected based upon the complete preoperatory assessment, independently of the study and in accordance with current clinical practice. All patients received routine postsurgical recommendations to participate in follow-up visits at the following postoperative intervals: 1 day, 1 week, 1 month, 6 months, and 1 year. The study selected patients that presented for the clinic-recommended follow-up appointments for at least one-month post-operation. For patients lost to follow-up, several contact efforts were made to recommend follow-up appointments at no cost or pressure to continue the study. At any time, patients could request to opt out of the study without affecting any postoperative follow-up appointments. From the initial 121 patients (230 eyes), 66 patients (126 eyes) returned for the 6-month postoperative follow-up, and 45 patients (85 eyes) returned for the 1-year follow-up. 

### 2.2. Preoperative Assessment

Preoperative assessment included complete clinical examination, uncorrected distance visual acuity (UDVA) and best corrected distance visual acuity (BCVA or CDVA), manifest refraction, refraction and keratometry measurements without and with pharmacological cycloplegia, corneal topographic and tomographic profile, calculated laser-ablation profile. The study collected extensive surgical data from the laser-assisted ablative procedure—such as applied laser-profile parameters (target in spherical and cylinder diopters, cylinder axis, central pachymetry, ablative targeted epithelial thickness (EPI for Trans-PRK), or targeted flap thickness (for FS-LASIK)—predicted residual stromal thickness, ablation zone size, optical and transition zone sizes, ablative MAX and CEN parameters, intraoperative pachymetry changes, laser-treatment total duration, number of breaks in the laser-treatment, and their duration (especially for Trans-PRK). Follow-up data included consequent clinical examinations, uncorrected distance visual acuity (UDVA) and best corrected distance visual acuity (BCVA or CDVA), manifest refraction, refraction and keratometry measurements, and corneal topographic and tomographic data.

### 2.3. Surgical Procedure

All surgical procedures were performed using the Alcon Wavelight^®^ Refractive Surgery System comprised of the Wavelight^®^ FS200^®^ 50 Hz, 193 nm femtosecond laser and the Wavelight^®^ EX500^®^ 500 Hz Ultraviolet (UV) excimer laser. Preoperative and postoperative data was collected using the corneal-tomograph and Scheimpflug camera Wavelight^®^ Oculyzer™ II and the corneal tomograph Wavelight^®^ Topolyzer™ Vario. Calculations were performed using Alcon wavefront measurement. Several cases also required the use of topography software Alcon Contoura software suite for topography-guided ablation versions SP3 (2019–2020), SP4 (2021–2022) and Green SP5 (late 2022). Surgery was performed using either Trans-PRK or FS-LASIK. The selected surgical technique was decided upon the complete surgical assessment and was independent of the study. Mitomycin-C 0.02% was routinely applied at the end of the surgery to all patients via timed application with surgical sponge. Ten Trans-PRK patients also underwent corneal crosslinking at the end of the surgery based on surgical assessment criteria.

### 2.4. Statistical Analysis

For statistical analysis, data were collected from both physical written patient records and the clinic’s electronic database into a deidentified EXCEL database (EXCEL 365 2023 Versions 2310-2311) (Microsoft Corporation, Redmond, WA, USA), where statistical analysis and graph-building was performed. Visual acuity data was measured using an Early Treatment of Diabetic Retinopathy Study Optotype (ETDRS Optotype) collected in decimal form from the patient records and was further converted into Snellen lines or LogMAR using a conversion chart. Visual acuity data were collected up to the value of 1.0 decimal (20/20 Snellen, logMAR 0). In the case of decimal recordings without exact correspondence to Snellen lines (such as 0.9 decimal in-between Snellen 20/25 and 20/20 or 0.7 decimal in-between 20/32 and 20/25), the nearest lower Snellen line was chosen (0.9 decimal was converted to 20/25 Snellen; 0.7 decimal to 20/32 and so onwards). LogMAR conversion from decimal values was performed for statistical analysis. Further statistical analysis was performed using Minitab^®^ 20.3 (64-bit) (© Minitab, LLC, State College, PA, USA), and the latest subscription-based version of IBM’s SPSS Statistics Software version 29 (International Business Machines Corporation, New York, NY, USA). For vectorial astigmatism calculations, the AstigMATIC [10] software version 2.0 for standard astigmatism vector analysis presented in the paper by Gauvin, M., Wallerstein, A. [10] was used. Definitions for terms in the vector analysis were as follows: target induced astigmatism (TIA) is the targeted astigmatic change, surgically induced astigmatism (SIA) is the astigmatic change recorded after surgery, difference vector (DV) is the difference between the SIA and TIA vectors and refers to residual postsurgical astigmatism, magnitude of error (ME) is the arithmetic difference between SIA and TIA, corrective index (CI) is the ratio of SIA to TIA and represents corrected astigmatism. CI ideally tends towards equaling one with values below one representing undercorrection and values over one overcorrection; CI can also be expressed as percentage (CI% or CI × 100%) reflecting the percentage of astigmatism correction achieved with values under 100% representing undercorrection and values above 100% overcorrection. 

### 2.5. Study Limitations

FS-LASIK can approach a larger diopter range, such as significantly extending useful hyperopic correction ranges or high astigmatism correction. Consequently, in accordance with the study’s non-randomized and clinical best practice context, the surgical technique predominantly chosen for hyperopic or higher astigmatism patients was FS-LASIK, while a higher percentage of myopic or myopic astigmatism patients were selected for the Trans-PRK technique. Thus, the Trans-PRK and FS-LASIK populations can present preoperative differences. Extensive preoperative data for each technique is presented in the results section.

The prospective design of the study, patient selection criteria and development in a single clinical practice limited the selected patients to 121 (total 230 eyes) initial patients; 66 patients (126 eyes) returned for the 6-month postoperative follow-up, and 45 patients (85 eyes) returned for the 1-year follow-up.

### 2.6. Local Ethics Committee Approval

The study and publishing of the study results were approved by the Local Ethics Committee for Scientific Research of the Clinic: Clinical Hospital for Ophthalmological Emergencies Bucharest (5893/29 November 2023). Following the analysis of the submitted study documents regarding patients from the Alcor Clinic, which include requests to use data pertaining to clinical evaluation for refractive surgery, such as information from clinical examination; slit-lamp images and video; measured visual acuity; and investigations including keratometric and refraction values, non-contact tonometry, corneal tomography and topography data, previous medical history, and surgical preoperative data including calculated laser-ablative profile parameters; and surgical data, such as laser-treatment parameters, duration and intraoperative images and video, postoperative follow-up data of the patients including clinical examinations, slit-lamp images and video, measured visual acuity, keratometric and refraction values, corneal tomography and topography, and other patient data, the Ethics Committee approved of the aforementioned publication of the study.

## 3. Results

### 3.1. Demographics and Preoperative Data

A total of 121 patients (230 eyes) were enrolled in the study population, with loss of follow-up at 6 months and 1 year, with 66 patients (126 eyes) returning for the 6-month postoperative follow-up and 45 patients (85 eyes) returning for the 1-year follow-up. Trans-PRK was performed for 87 patients—167 eyes and FS-LASIK for 34 patients—63 eyes. The mean patient age was generally 26.683 years (standard deviation (StDev) 8.879)—25.235 years (7.757 StDev) for Trans-PRK and 30.3 years (Stdev 10.45) for FS-LASIK. Mean preoperative CDVA for all patients was 0.92609 decimal ± 0.14722 or 0.04913 logMAR ± 0.09808. Trans-PRK mean preoperative CDVA was 0.95269 decimal ± 0.11397 or 0.03246 logMAR ± 0.08008, while FS-LASIK mean preoperative CDVA was 0.8556 decimal ± 0.1957 or 0.0933 logMAR ± 0.1250. Preoperative Trans-PRK refraction was −2.741 spherical diopters (SPH D) and −1.462 cylinder diopters (CYL D), with a recorded minimum of −11 SPH D and −7 CYL D and maximum of +5.25 SPH D and +1.25 CYL D. For FS-LASIK, preoperative refraction was +1.556 spherical diopters (SPH D) and −1.111 cylinder diopters (CYL D), with a recorded minimum of −9 SPH D and −6.25 CYL D and maximum of +7D SPH and +5 CYL D. Mean ablative spherical target was −2.722 SPH D ± 2.696 for Trans-PRK and +0.944 SPH D ± 3.668 for FS-LASIK. Mean ablative astigmatism correction was −1.178 CYL D ± 1.557 for Trans-PRK and +0.214 CYL D ± 2.886 for FS-LASIK. Further preoperative data, such as cycloplegia refraction, best corrected visual acuity diopters, and min/max ablative target, are included in Table 1. Attempted correction is defined by the Attempted Spherical Equivalent (SE)—the calculated SE representing D SPH + ½ x D CYL from the laser target parameters. The mean attempted SE is positive for FS-LASIK at +1.052 (StDev 4.109) and negative for Trans-PRK at −3.311 D (StDev 2.690). However, to better represent the corrective power demanded from each surgical technique, an absolute (+Sign) attempted spherical equivalent was calculated for each recording and subsequently an average of only the absolute attempted SE produced. In average Trans-PRK was requested to correct a mean absolute attempted SE of 3.778 D (1.977 StDev) versus a similar 3.710 D (2.005 StDev) value for FS-LASIK. Normality testing using the Ryan–Joiner and Kolmogorov–Smirnov revealed normally distributed data as follows for preoperative visual acuity and refraction data (such as refraction SPH and CYL D, cycloplegic refraction SPH and CYL D, CDVA SPH and CYL D, ablative target in SPH and CYL D, ablative target in SE D) and postoperative visual acuity and refractive data (visual acuity UCVA, CDVA, post-op refraction SPH and CYL D, SE D for post-op 1 day, 1 week, 1 month, 6 months and 1 year). 

### 3.2. Surgical Data and Postoperative Results

Ablative parameters are at large presented in Table 2 for each procedure—Trans-PRK or FS-LASIK—and for the entire study population (overall, both procedures). Statistical comparison between the Trans-PRK and FS-LASIK populations using two-sample *t*-test is presented in Table 3. 

**Figure 2:**
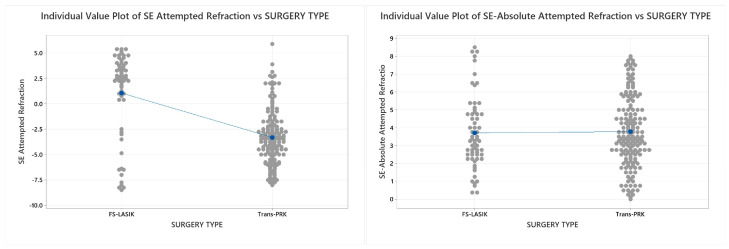
Individual value plots comparing the Trans-PRK and FS-LASIK value distributions for attempted spherical equivalent (SE) (different for the study populations) and absolute attempted SE (comparable for both Trans-PRK and FS-LASIK populations). The blue line connects the mean values of the FS-LASIK and Trans-PRK populations. Trans-PRK: transepithelial photorefractive keratectomy; FS-LASIK: femtosecond-laser-assisted in situ keratomileusis.

The Trans-PRK and FS-LASIK populations present statistical difference in the ablative target in spherical diopters (Trans-PRK −2.722 ± 2.696 versus FS-LASIK +0.944 ± 3.668 D; *p* < 0.001), ablative target in cylinder diopters (Trans-PRK −1.178 ± 1.557 versus FS-LASIK +0.214 ± 2.886 D; *p* = 0.001), and attempted Spherical equivalent (Trans-PRK −3.311 ± 2.69 versus FS-LASIK +1.052 ± 4.109 D; *p* = 0.001). Despite this, comparing the mean of absolute attempted SE yielded no statistical difference and a similar demand of corrective power (Trans-PRK 3.778 ± 1.977 versus FS-LASIK 3.710 ± 2.005 D; *p* = 0.82). Figure 2 represents a graphical correspondence of Attempted SE—Absolute attempted SE. 

Furthermore, examining the early postoperative period of Trans-PRK versus FS-LASIK (Table 3) only PostOp 1 Day SE differs statistically (Trans-PRK −0.129 versus −0.521 for FS-LASIK, *p* = 0.043) with PostOp 1 Day SPH D and CYL D not presenting a statistical difference (*p* = 0.089 and 0.39 respectively). At 1 week and 1 month post-operation, no significant difference was recorded in the study population regarding chosen surgical technique. At later postoperative follow-up at 6 months, a difference was found for spherical diopters (Trans-PRK −0.0476 ± 0.7012 versus FS-LASIK +0.425 ± 0.874, *p* = 0.004) and spherical equivalent (Trans-PRK −0.1994 ± 0.0294 versus FS-LASIK +0.225 ± 0.646, *p* = 0.025) but not for CYL D (Trans-PRK −0.3036 ± 0.5251 versus FS-LASIK −0.4 ± 0.820, *p* = 0.499). This resulted in better uncorrected visual acuity (UCVA Decimal and logMAR) for Trans-PRK patients at 6 months post-operation (UCVA logMAR 0.02523 Decimal 0.9602 Trans-PRK versus 0.0768 logMAR and 0.8833 decimal for FS-LASIK; *p* = 0.021 UCVA Decimal, respectively *p* = 0.015 logMAR). The statistical differences did not persist towards the 1-year follow-up for UCVA Decimal (*p* = 0.079) and logMAR (*p* = 0.072) or CYL D (*p* = 0.286) but did persist for spherical diopters (Trans-PRK −0.0294 ± 0.6493 versus FS-LASIK +0.646 ± 0.909, *p* < 0.001) and spherical equivalent (Trans-PRK −0.218 ± 0.784 versus FS-LASIK 0.372 ± 1.08, *p* = 0.007). Analysis of variance testing (ANOVA) revealed a difference in variance of measurements between post-op 6-months Trans-PRK and FS-LASIK patients (SPH D *p* = 0.002, SE *p* = 0.013, UCVA Decimal *p* = 0.006, UCVA logMAR *p* = 0.004) and between post-op 1-year Patients (SPH D *p* < 0.001, SE *p* = 0.004, UCVA logMAR *p* = 0.045 but not for UCVA Decimal *p* = 0.061) with FS-LASIK population having slightly higher variance in measurements.

The results are summarized in the standardized refractive surgery graphs represented in Figure 3, Figure 4, Figure 5, Figure 6, Figure 7, Figure 8, Figure 9, Figure 10 and Figure 11.

### 3.3. Astigmatism Calculation

For vectorial astigmatism calculations, the AstigMATIC [10] software for standard astigmatism vector analysis presented in the paper by Gauvin, M. and Wallerstein, A. [10] was used for Trans-PRK (Figure 12) and FS-LASIK (Figure 13) (AstigMATIC version 2.0). Target-induced astigmatism (TIA) is the targeted astigmatic change, surgically induced astigmatism (SIA) is the astigmatic change recorded after surgery, difference vector (DV) is the difference between the SIA and TIA vectors and refers to residual postsurgical astigmatism, magnitude of error (ME) is the arithmetic difference between SIA and TIA, refractive angle of error is the difference between the SIA axis and TIA axis; corrective index (CI) is the ratio of SIA to TIA and represents corrected astigmatism. CI ideally tends towards equaling one with values below one representing undercorrection and values over one overcorrection; CI can also be expressed as percentage (CI% or CI × 100%) reflecting the percentage of astigmatism correction achieved with values under 100% representing undercorrection and values above 100% overcorrection. 

To simplify the presentation of the data from vectorial astigmatism calculation it has been included into Table 4. Overall FS-LASIK presented higher TIA and SIA astigmatism across the board at different postoperative intervals. The difference vector was higher for FS-LASIK apart from 1 week postoperative, while the corrective index was overall slightly higher for Trans-PRK.

### 3.4. Safety and Efficacy

**Figure 3:**
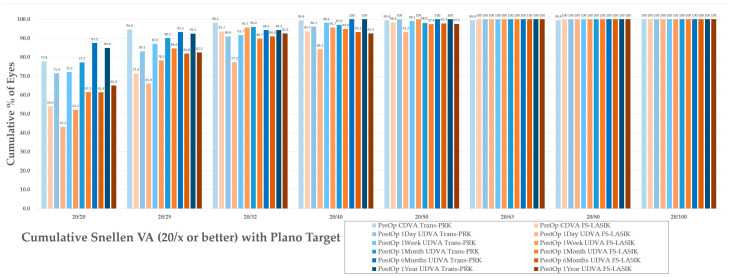
Cumulative Snellen visual acuity (VA) with Plano target by treatment type. Trans-PRK: transepithelial photorefractive keratectomy; FS-LASIK: femtosecond-laser-assisted in situ keratomileusis; CDVA—corrected distance visual acuity; UDVA—uncorrected distance visual acuity.

**Figure 4:**
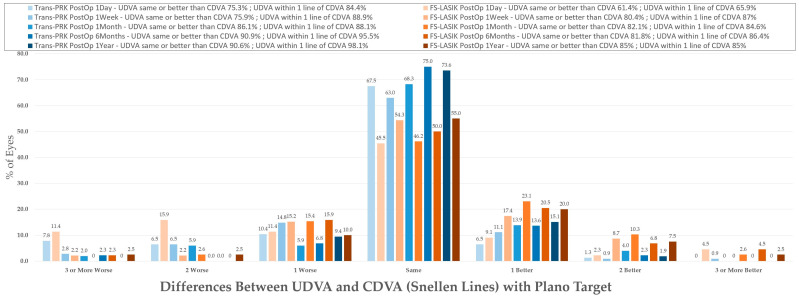
Differences between uncorrected distance visual acuity (UDVA) and corrected distance visual acuity type (CDVA) Snellen lines with plano target. Trans-PRK: transepithelial photorefractive keratectomy; FS-LASIK: femtosecond-laser-assisted in situ keratomileusis.

For evaluation of the safety index, the percentage of patients that lost 2 or more lines was calculated (Figure 4) at between 0–2.3% for Trans-PRK over the 1-week–1-year postoperative period and at 0–4.3% for FS-LASIK over the 1-week–1-year postoperative period (Figure 4). No Trans-PRK patients from the 1-year follow-up lost two or more lines of CDVA. Overall, the safety index was thus higher than the reported 0.85 (85%) cut-off level [11]. Efficacy refers to the ratio of preoperative BCVA to postoperative UCVA and presents an 0.8 (80%) cut-off [11] corresponding to two or fewer lines of visual acuity. In the study, one line of difference was used as a higher cutoff, resulting in 88.9–98.1% of Trans-PRK patients and 85–87% of FS-LASIK within one line of CDVA (Figure 3). Overall, in the study, both procedures were within safety and efficacy indexes, with Trans-PRK presenting slightly better results.

### 3.5. Encountered Postoperative Complications

For Trans-PRK in the earlier postoperative period, two patients noted significant postoperative pain which subsequently resolved under analgetic treatment. Mild to severe dry eye syndrome was encountered in five patients, with one patient requiring the application of a therapeutic contact lens for 1 month. Corneal haze was encountered in four patients at the 6-month follow-up; only two patients presented symptomatic decrease in visual acuity and required treatment with topical fluorometholone which improved visual acuity after 1 month. One of the other two non-symptomatic patients presented only mild peripheric haze in one eye and otherwise had an excellent visual acuity of Snellen 20/12.5 in both eyes. One patient presented microcystic degenerescence at 1 and 2 months post-operation, which receded with further time and treatment.

For FS-LASIK, severe dry eye syndrome was encountered 2 months post-operation. Rainbow glare was encountered in 1 patient, with the glare slightly improving in subsequent months, but did not disappear at the latest 1-year follow-up. Subepithelial haze was reported 6 months post-operation for one patient. Myopic or hyperopic shift in refraction (after 2–6 months post-operation) was noted in two patients, one shifted myopic and the other hyperopic. Optical correction improved visual acuity to preoperative levels. No major surgical complications, such as corneal ectasia disease, infectious complications, or persistent severe dry eye disease, were encountered. For femto-LASIK the only flap-related complications were one rainbow-glare and one subepithelial haze formation, with the patients expected to return for later follow-up and therapeutical management. Otherwise, the created flaps were mechanically stable and underwent normal healing processes.

## 4. Discussion

**Figure 7:**
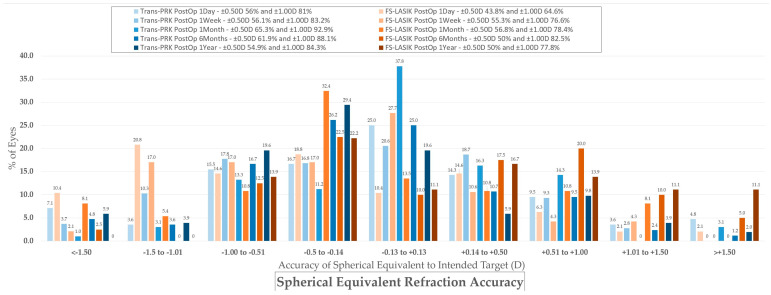
Spherical equivalent refraction accuracy. Trans-PRK: transepithelial photorefractive keratectomy; FS-LASIK: femtosecond-laser-assisted in situ keratomileusis.

**Figure 12:**
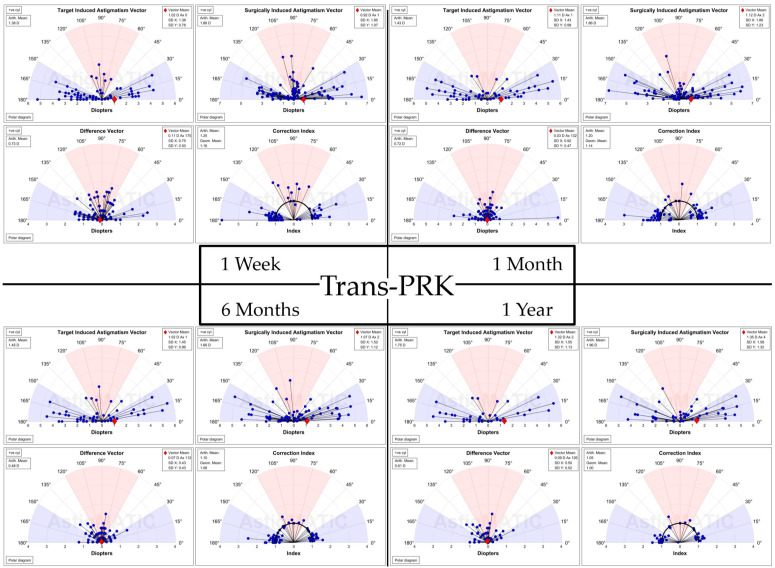
Vectorial astigmatism calculations for Trans-PRK using AstigMATIC [10] (version 2.0). The colored regions of the graph represent: red for with-the-rule astigmatism (WTR), white for oblique astigmatism and blue for against-the-rule astigmatism (ATR) [10]. Individual vectors are represented as a black line ending in a blue circle. The red diamond indicates the mean vector position [10].

**Figure 13:**
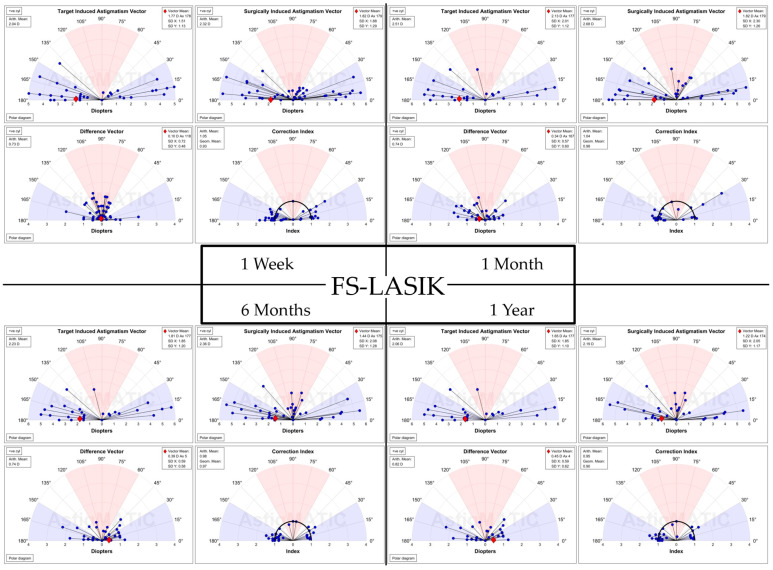
Vectorial astigmatism calculations for FS-LASIK using AstigMATIC [10] (version 2.0). The colored regions of the graph represent: red for with-the-rule astigmatism (WTR), white for oblique astigmatism and blue for against-the-rule astigmatism (ATR) [10]. Individual vectors are represented as a black line ending in a blue circle. The red diamond indicates the mean vector position [10].

Our study evaluated the results from Trans-PRK and FS-LASIK in a single-clinic, non-randomized context, selecting the surgical technique based on established best practice norms. Although FS-LASIK presents more established surgical correction potential, especially for hyperopia, the two techniques have overlapping indications and could either be used for most cases. Trans-PRK, present as “Streamlight” on the refractive platform used in the study represents an overall improvement in visual recovery and epithelial healing over the initial PRK technique [3]. Chang JY. et al. [12] highlighted the relative inconclusiveness of studies comparing Trans-PRK and LASIK. On one hand, Trans-PRK was reported to provide better or comparable results for myopic patients [12,13] and could provide a lower increase in corneal higher order aberrations versus FS-LASIK [14]. However, other studies suggest more caution, with Gershoni A. et al. [15] reporting better result with FS-LASIK for low–moderate-grade myopia and Sabhapandit S. et al. [16] highlighting hyperopia as an uncharted territory for Trans-PRK [16]. In this study, despite the initial differences in the study population for Trans-PRK and FS-LASIK leading towards a myopic population for Trans-PRK and a slightly hyperopic one for FS-LASIK (Ablative target in spherical diopters (Trans-PRK −2.722 ± 2.696 versus FS-LASIK +0.944 ± 3.668 D; *p* < 0.001), ablative target in cylinder diopters (Trans-PRK −1.178 ± 1.557 versus FS-LASIK +0.214 ± 2.886 D; *p* = 0.001) and attempted spherical equivalent (Trans-PRK −3.311 ± 2.69 versus FS-LASIK +1.052 ± 4.109 D; *p* = 0.001), the early postoperative results at 1 week and 1 month did not present notable statistical differences. In accordance with Figure 7 highlighting the spherical equivalent refraction stability, a slight trend towards initial overcorrection by both techniques can be hypothesized. For FS-LASIK, this overcorrection receded towards a slight under-correction at 6 months and 1 year post-operation; for Trans-PRK, this phenomenon was not as notable. Visual acuity was notably better in the Trans-PRK group in the first postoperative day (*p* = 0.003 for decimal and *p* = 0.001 for logMAR), thus suggesting a faster visual recovery versus FS-LASIK. Visual acuity remained higher in the Trans-PRK population at later follow-up. This resulted in better uncorrected visual acuity (UCVA Decimal and logMAR) for Trans-PRK patients 6 months post-operation (UCVA logMAR 0.02523 Decimal 0.9602 Trans-PRK versus 0.0768 logMAR and 0.8833 decimal for FS-LASIK; *p* = 0.021 UCVA Decimal, respectively *p* = 0.015 logMAR). The statistical differences did not persist towards the 1-year follow-up for UCVA Decimal (*p* = 0.079) and logMAR (*p* = 0.072) or CYL D (*p* = 0.286) but did persist for spherical diopters (Trans-PRK −0.0294 ± 0.6493 versus FS-LASIK +0.646 ± 0.909, *p* < 0.001) and spherical equivalent (Trans-PRK −0.218 ± 0.784 versus FS-LASIK 0.372 ± 1.08, *p* = 0.007). Overall, Trans-PRK seemed to offer more precise spherical correction and more consistent visual results, as highlighted by ANOVA differences between groups in favor of Trans-PRK and of higher calculated surgical induced astigmatism parameters TIA and SIA across the board at different postoperative intervals. The difference vector was higher for FS-LASIK apart from 1 week post-operation, while the corrective index was overall slightly higher for Trans-PRK.

Trans-PRK is a highly useful and adaptive technique, allowing for combination with cross-linking in more difficult cases to provide increased corneal biomechanical stability and presents use for topographical ablation procedures [17]. Apart from the refractive platform used in the study, the technique’s availability is increasing, with other platforms offering it in wavefront-optimized or wavefront-guided forms [18]. For thinner corneas, Trans-PRK significantly used up a reduced amount of corneal stroma (mean predicted residual stroma 430.10 µm versus 362.46 µm for LASIK) while correcting a similar amount of absolute spherical equivalent diopters (3.778 Trans-PRK versus 3.710 FS-LASIK). MAX stromal excision parameter was reduced in Trans-PRK: 65.87 µm Trans-PRK MAX versus 78.79 µm FS-LASIK MAX; however, CEN was increased 60.93 µm Trans-PRK CEN versus 25.16 µm FS-LASIK CEN. Excimer laser-treatment times were increased due to adding the epithelial step from a mean 16.75 s for FS-LASIK to 45.988 s for Trans-PRK with a higher number of breaks needed 1.5509 versus 0.317 for FS-LASIK; the higher number of breaks also respected operative protocol for prevention of increased corneal thermal load [19]. Nevertheless, if we also consider the femtosecond flap creation required before LASIK stromal ablation, Trans-PRK offers a convenient and effective single-laser solution. 

Events encountered post-operation included sporadic cases of dry eye syndrome and corneal haze for both procedures. Two patients in the Trans-PRK group noted significant postoperative pain that responded favorably to analgetic administration and subsequently resolved. For FS-LASIK, one case of rainbow-glare was reported with the symptoms reducing but not ceasing 1 year post-operation. All patients received specific treatment and further follow-up visits and remain in active follow-up. Calculated safety and efficacy parameters were within literature-reported standards throughout the study [11]. 

## 5. Conclusions

Our study reported improvements in first-day visual recovery, late postoperative visual acuity, spherical refraction, and spherical equivalent for patients undergoing Trans-PRK versus FS-LASIK. However, both FS-LASIK and Trans-PRK presented good overall results and achievement of refractive surgery goals. For patients suitable for either technique, Trans-PRK could offer better results; furthermore, the availability of Trans-PRK is increasing with multiple refractive surgery platforms offering the technique. 

## Figures and Tables

**Figure 5:**
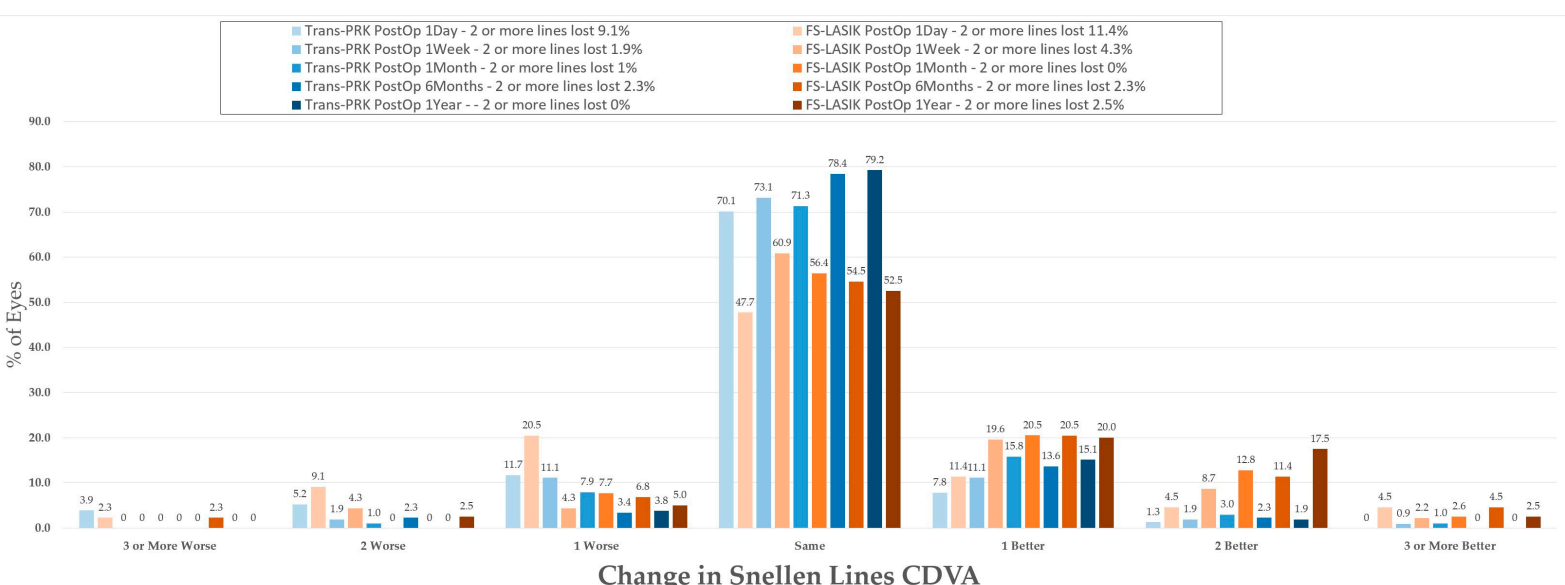
Changes in corrected distance visual acuity type (CDVA) Snellen lines. Trans-PRK: transepithelial photorefractive keratectomy; FS-LASIK: femtosecond-laser-assisted in situ keratomileusis.

**Figure 6:**
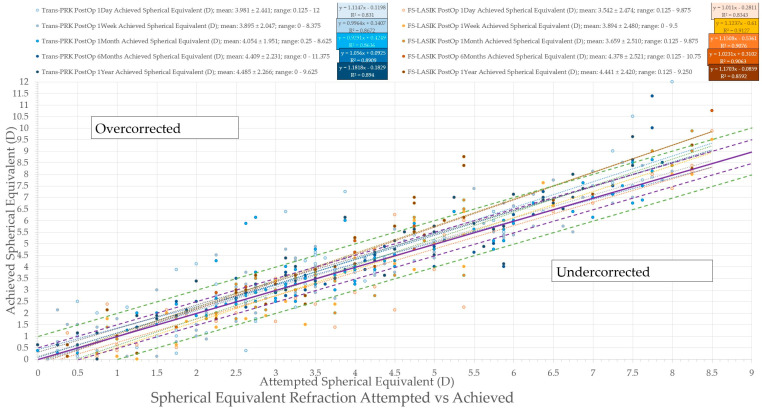
Spherical equivalent refraction—attempted vs. achieved spherical equivalent refractions. The purple solid line represents the equation x = y and an ideal result; the purple dashed line represents ± 0.5 diopter (D), and the green dashed line ± 1 diopter (D) of the ideal line. Trans-PRK: transepithelial photorefractive keratectomy; FS-LASIK: femtosecond-laser-assisted in situ keratomileusis.

**Figure 8:**
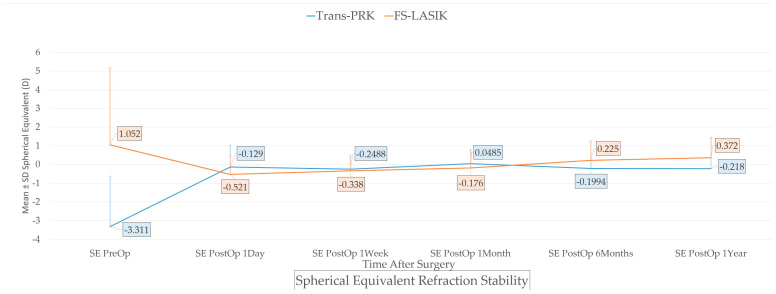
Spherical equivalent refraction stability. Trans-PRK: transepithelial photorefractive keratectomy; FS-LASIK: femtosecond-laser-assisted in situ keratomileusis.

**Figure 9:**
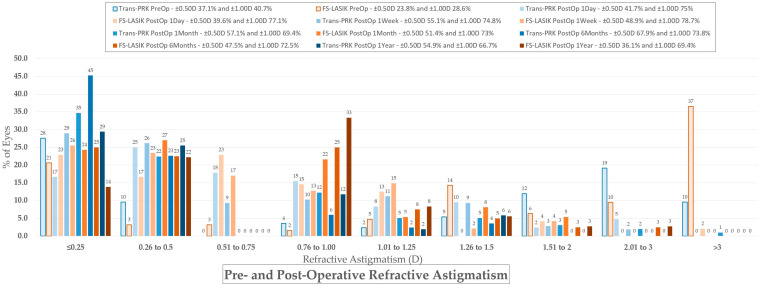
Change in refractive astigmatism. Trans-PRK: transepithelial photorefractive keratectomy; FS-LASIK: femtosecond-laser-assisted in situ keratomileusis.

**Figure 10:**
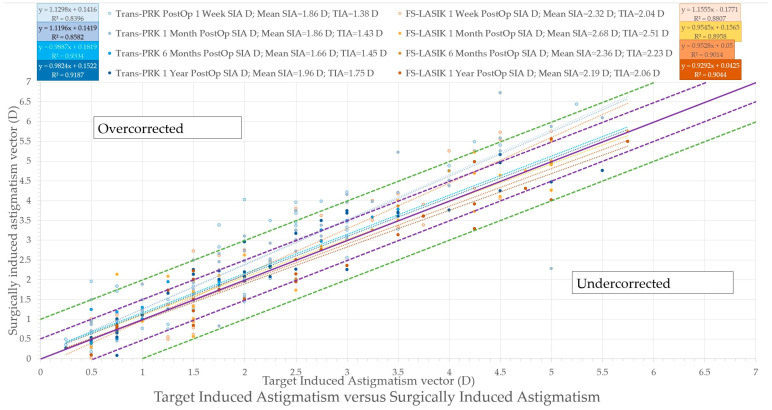
Target-induced astigmatism (TIA) versus surgically induced astigmatism (SIA). The purple solid line represents the equation x = y and an ideal result; the purple dashed line represents ± 0.5 diopter (D), and the green dashed line represents ± 1 diopter (D) of the ideal line. Trans-PRK: transepithelial photorefractive keratectomy; FS-LASIK: femtosecond-laser-assisted in situ keratomileusis.

**Figure 11:**
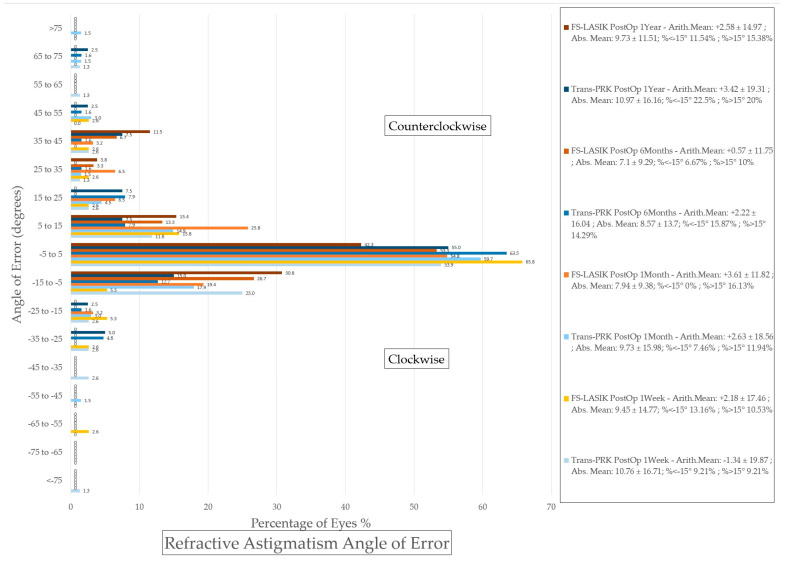
Histogram of refractive astigmatism angle of error. Trans-PRK: transepithelial photorefractive keratectomy; FS-LASIK: femtosecond-laser-assisted in situ keratomileusis.

**Table 1:** Demographics and preoperative data.

Categories	Trans-PRK	FS-LASIK	Both Procedures
Total eyes (n)	167	63	230
Patient Age (years)	25.235	30.3	26.683
Patient Age StDev	7.757	10.45	8.879
PreOp CDVA decimal (Mean)	0.95269	0.8556	0.92609
PreOp CDVA decimal (SE Mean)	0.00882	0.0247	0.00971
PreOp CDVA decimal (StDev)	0.11397	0.1957	0.14722
PreOp CDVA decimal (Minimum)	0.3	0.2	0.2
PreOp CDVA decimal (Median)	1	1	1
PreOp CDVA decimal (Maximum)	1	1	1
PreOp CDVA logMAR (Mean)	0.3246	0.0933	0.04913
PreOp CDVA logMAR (SE Mean)	0.00620	0.0157	0.00647
PreOp CDVA logMAR (StDev)	0.08008	0.1250	0.09808
PreOp CDVA logMAR (Minimum)	0.7	0.5	0.7
PreOp CDVA logMAR (Median)	0	0	0
PreOp CDVA logMAR (Maximum)	0	0	0
PreOp Refraction Mean SPH D	−2.741	+1.556	−1.554
PreOp Refraction Mean CYL D	−1.462	−1.111	−1.365
PreOp Refraction StDev SPH D	2.919	4.483	3.918
PreOp Refraction StDev CYL D	1.393	2.973	1.960
PreOp Refraction Minimum SPH D	−11	−9	−11
PreOp Refraction Maximum SPH D	+5.250	+7	+7
PreOp Refraction Minimum CYL D	−7	−6.25	−7
PreOp Refraction Maximum CYL D	+1.250	+5	+5
PreOp Cy. Mean Refraction SPH D	−2.375	+1.980	−1.171
PreOp Cy. Mean Refraction CYL D	−1.375	−0.687	−1.184
PreOp Cy. Refraction StDev SPH D	3.014	4.536	3.999
PreOp Cy. Refraction StDev CYL D	1.494	3.016	2.050
PreOp Cy. Refraction Minimum SPH D	−9.25	−8.75	−9.250
PreOp Cy. Refraction Maximum SPH D	+6	+7.75	+7.750
PreOp Cy. Refraction Minimum CYL D	−6.5	−6.5	−6.500
PreOp Cy. Refraction Maximum CYL D	+2.5	+5.75	+5.750
PreOp CDVA Mean SPH D	−2.758	+1.213	−1.652
PreOp CDVA Mean CYL D	−1.113	−0.107	−0.822
PreOp CDVA StDev SPH D	2.520	3.910	3.459
PreOp CDVA StDev CYL D	1.573	2.828	2.061
PreOp CDVA Minimum SPH D	−8.25	−8.5	−8.500
PreOp Cy.CDVA Maximum SPH D	+3	+6.5	+6.500
PreOp Cy. CDVA Minimum CYL D	−5.5	−5.75	−5.750
PreOp Cy. CDVA Maximum CYL D	+4	+4.5	4.500
Ablative Target Mean SPH D	−2.722	+0.944	−1.717
Ablative Target StDev SPH D	2.696	3.668	3.405
Ablative Target Mean CYL D	−1.178	+0.214	−0.797
Ablative Target StDev CYL D	1.557	2.886	2.097
Ablative Target Minimum SPH D	−8	−7.5	−8
Ablative Target Maximum SPH D	+6.25	+5	+6.25
Ablative Target Minimum CYL D	−5.5	−5.75	−5.75
Ablative Target Maximum CYL D	+3	+5	+5

Demographics and preoperative best corrected visual acuity (CDVA) and refractive data. PreOp—preoperative; SE Mean—standard error mean; StDev—standard deviation; SPH D—spherical diopters; CYL D—cylinder diopters; Cy.—cycloplegia.

**Table 2:** Ablation target parameters (laser-treatment target parameters) and intraoperative data, such as treatment duration and number of breaks.

Categories	Trans-PRK	FS-LASIK	Both
Total eyes (n)	167	63	230
Ablative Target Mean SPH D	−2.722	+0.944	−1.717
Ablative Target StDev SPH D	2.696	3.668	3.405
Ablative Target Mean CYL D	−1.178	+0.214	−0.797
Ablative Target StDev CYL D	1.557	2.886	2.097
Ablative Target Minimum SPH D	−8	−7.5	−8
Ablative Target Maximum SPH D	+6.25	+5	+6.25
Ablative Target Minimum CYL D	−5.5	−5.75	−5.75
Ablative Target Maximum CYL D	+3	+5	+5
Mean PreOp Central Pachymetry µm	547.06	558.70	550.25
PreOp Min. Central Pachymetry µm	473	493	473
PreOp Max. Central Pachymetry µm	614	627	627
Mean EPI/FLAP Thickness µm	51.078	117.44	69.26
Mean Predicted Residual Stroma µm	430.10	362.46	411.57
Predicted Min. Residual Stroma µm	335	315	315
Predicted Max. Residual Stroma µm	533	439	533
Mean Ablative Mean Pupil Size mm	6.5	6.5	6.5
Mean Ablative Zone Size mm	8.4611	8.8762	8.5748
Mean Ablative Transition Zone Size mm	1.0428	1.2119	1.0891
Mean Ablative Optical Zone Size mm	6.4	6.4603	6.4165
Mean Ablative MAX Parameter µm	65.87	78.79	69.41
Mean Ablative CEN Parameter µm	60.93	25.16	51.13
Mean Intraoperative Central Pachymetry mm	566.56	556.4	562.27
Mean Intraoperative EPI/FLAP-OFF mm	445	443	443.07
Mean Total Laser-Treatment Duration s	45.988	16.75	37.98
Mean Number of Breaks s	1.5509	0.317	1.213
Mean Cumulative Break Time s	15.234	2.190	11.661
Mean Attempted Refraction—SE Target D	−3.311	+1.052	−2.116
Mean Absolute Attempted Refraction (SE Absolute D)	3.778	3.710	3.759

Ablation target parameters D—diopters, SPH D—spherical diopters; CYL D—cylinder diopters; EPI—epithelial removal in µm; FLAP—flap thickness for FS-LASIK in µm; MAX and CEN—surgical parameters for stromal ablation; SE—spherical equivalent.

**Table 3:** Preoperative versus postoperative refractive and visual acuity data.

Categories	Trans-PRK	FS-LASIK	*p* Value Trans-PRK vs. FS-LASIK	All Patients
Ablative Target Mean SPH D	−2.722	+0.944	<0.001	−1.717
Ablative Target StDev SPH D	2.696	3.668		3.405
Ablative Target Mean CYL D	−1.178	+0.214	0.001	−0.797
Ablative Target StDev CYL D	1.557	2.886		2.097
Attempted Refraction—Mean SE Target	−3.311	+1.052	0.000	−2.116
Attempted Refraction—Mean SE StDev	2.690	4.109		3.690
Mean of Absolute Attempted SE	3.778	3.710	0.820	
Attempted SE in Absolute Value StDev	1.977	2.005		
PostOp 1 Day—Refraction SPH D	+0.167	−0.156	0.089	+0.0492
PostOp 1 Day—Refraction SPH StDev	1.067	1.022		1.0582
PostOp 1 Day—Refraction CYL D	−0.5923	−0.729	0.390	−0.6420
PostOp 1 Day—Refraction CYL StDev	0.8562	0.889		0.8672
PostOp 1 Day—SE	−0.129	−0.521	0.043	−0.2718
PostOp 1 Week—Refraction SPH D	+0.0304	−0.0798	0.350	−0.0032
PostOp 1 Week—Refraction SPH StDev	0.6647	0.6718		0.6667
PostOp 1 Week—Refraction CYL D	−0.5584	−0.516	0.731	−0.5455
PostOp 1 Week—Refraction CYL StDev	0.7093	0.7		0.7045
PostOp 1 Week—SE	−0.2488	−0.338	0.487	−0.2760
PostOp 1 Month—Refraction SPH D	+0.3061	+0.041	0.058	+0.2333
PostOp 1 Month– Refraction SPH StDev	0.7877	0.683		0.7673
PostOp 1 Month—Refraction CYL D	−0.5152	−0.432	0.599	−0.4925
PostOp 1 Month—Refr. CYL StDev	0.8838	0.783		0.8553
PostOp 1 Month—SE	+0.0485	−0.176	0.150	−0.0129
PostOp 6 Months—Refraction SPH D	−0.0476	+0.425	0.004	+0.1048
PostOp 6 Months—Refr. SPH StDev	0.7012	0.874		0.7893
PostOp 6 Months—Refraction CYL D	−0.3036	−0.400	0.499	−0.3347
PostOp 6 Months—Refr. CYL StDev	0.5251	0.820		0.6335
PostOp 6 Months—SE	−0.1994	+0.225	0.025	−0.0625
PostOp 1 Year—Refraction SPH D	−0.0294	+0.646	<0.001	0.25
PostOp 1 Year—Refraction SPH StDev	0.6493	0.909		0.8327
PostOp 1 Year—Refraction CYL D	−0.3775	−0.549	0.286	−0.4483
PostOp 1 Year—Refraction CYL StDev	0.6049	0.808		0.6969
PostOp 1 Year—SE	−0.218	0.372	0.007	+0.026
PreOp CDVA Decimal	0.95269	0.8556	<0.001	0.92609
PostOp 1 Day UCVA Decimal	0.9130	0.7864	0.003	0.8669
PostOp 1 Week UCVA Decimal	0.9167	0.8652	0.093	0.9013
PostOp 1 Month UCVA Decimal	0.9446	0.8949	0.127	0.9307
PostOp 6 Months UCVA Decimal	0.9602	0.8833	0.021	0.9354
PostOp 1 Year UCVA Decimal	0.9528	0.8895	0.079	0.9264
PreOp CDVA logMAR	0.03246	0.0933	0.001	0.04913
PostOp 1 Day UCVA logMAR	0.06	0.1386	0.001	0.0886
PostOp 1 Week UCVA logMAR	0.05278	0.0817	0.125	0.06143
PostOp 1 Month UCVA logMAR	0.04277	0.728	0.176	0.05114
PostOp 6 Months UCVA logMAR	0.02523	0.0768	0.015	0.04242
PostOp 1 Year UCVA logMAR	0.0287	0.0720	0.061	0.0473

PostOp—postoperative; D—diopters, SPH D—spherical diopters; CYL D—cylinder diopters; CDVA—best corrected visual acuity; UCVA—uncorrected visual acuity. PreOp—preoperative; StDev—standard deviation; *p* Value was obtained using the two-sample *t*-test.

**Table 4:** Vectorial astigmatism results – mean values for each study population by surgery type (Trans-PRK and FS-LASIK) at follow-up intervals and calculated arithmetical difference between the means.

Time PostOp	Category	Trans-PRK	FS-LASIK	Diff.
1 Week	TIA arithmetic mean	1.38 D	2.04 D	0.66 D
1 Week	TIA mean vector	1.02 D Axis 0°	1.77 D Axis 178°	
1 Week	SIA arithmetic mean	1.86 D	2.32 D	0.46 D
1 Week	SIA mean vector	0.92 D Axis 1°	1.82 D Axis 179°	
1 Week	Difference vector arith. mean	0.73 D	0.73 D	No diff.
1 Week	Difference vector geom. mean	0.11 D Axis 170°	0.10 D Axis 118°	
1 Week	Correction Index arith. mean	1.25	1.05	0.2
1 Week	Correction Index geom. mean	1.16	0.93	0.23
1 Month	TIA arithmetic mean	1.43 D	2.51 D	1.08 D
1 Month	TIA mean vector	1.11 D Axis 1°	2.13 D Axis 177°	
1 Month	SIA arithmetic mean	1.86 D	2.68 D	0.82 D
1 Month	SIA mean vector	1.12 D Axis 2°	1.82 D Axis 179°	
1 Month	Difference vector arith. mean	0.72 D	0.74 D	0.02 D
1 Month	Difference vector geom. mean	0.03 D Axis 132°	0.34 D Axis 167°	
1 Month	Correction Index arith. mean	1.20	1.04	0.16
1 Month	Correction Index geom. mean	1.08	0.98	0.1
6 Months	TIA arithmetic mean	1.45 D	2.23 D	0.78 D
6 Months	TIA mean vector	1.02 D Axis 1°	1.81 D Axis 177°	
6 Months	SIA arithmetic mean	1.66 D	2.36 D	0.7 D
6 Months	SIA mean vector	1.07 D Axis 2°	1.44 D Axis 175°	
6 Months	Difference vector arith. mean	0.48 D	0.74 D	0.26 D
6 Months	Difference vector geom. mean	0.07 D Axis 112°	0.39 D Axis 5°	
6 Months	Correction Index arith. mean	1.1	0.98	0.12
6 Months	Correction Index geom. mean	1.08	0.97	0.11
1 Year	TIA arithmetic mean	1.75 D	2.06 D	0.31 D
1 Year	TIA mean vector	1.32 D Axis 2°	1.65 D Axis 177°	
1 Year	SIA arithmetic mean	1.96 D	2.19 D	0.23 D
1 Year	SIA mean vector	1.35 D Axis 4°	1.22 D Axis 174°	
1 Year	Difference vector arith. mean	0.61 D	0.82 D	0.21 D
1 Year	Difference vector geom. mean	0.09 D Axis 126°	0.45 D Axis 4°	
1 Year	Correction Index arith. mean	1.05	0.95	0.1
1 Year	Correction Index geom. mean	1.00	0.90	0.1

Vectorial astigmatism calculations. Diff—difference. PostOp—postoperative. Target induced astigmatism (TIA) is the targeted astigmatic change, surgically induced astigmatism (SIA) is the astigmatic change recorded after surgery, difference vector (DV) is the difference between the SIA and TIA vectors and refers to residual postsurgical astigmatism, magnitude of error (ME) is the arithmetic difference between SIA and TIA, corrective index (CI) is the ratio of SIA to TIA and represents corrected astigmatism. CI with values below one representing undercorrection and values over one overcorrection; CI can also be expressed as percentage (CI% or CI × 100%) reflecting the percentage of astigmatism correction achieved with values under 100% representing undercorrection and values above 100% overcorrection.

## Data Availability

All data regarding the study population pertains to Alcor Clinic Bucharest and is available upon request.
